# Naringenin Attenuates Diabetic Cardiac Injury via SIRT1 Activation in Streptozotocin‐Induced Rats

**DOI:** 10.1155/cdr/9581618

**Published:** 2026-10-09

**Authors:** Baolan Wang, Dandan Zhang, Fudan Zhang, Xue Meng, Li Zhang, Zhenzuo Li, Amirabas Bostani

**Affiliations:** ^1^ Department of Endocrinology The Fourth People′s Hospital of Jinan Affiliated to Shandong Second Medical University Jinan China; ^2^ Department of Biology Science & Research Institute Islamic Azad University Tehran Iran tiau.ac.ir

**Keywords:** cardioprotection, fibrosis, inflammation, oxidative stress, streptozotocin

## Abstract

**Objective:**

Diabetic cardiomyopathy (DCM) lacks targeted therapies. This study investigated naringenin (NAR), a citrus flavonoid, in a streptozotocin (STZ)‐induced diabetic rat model with cardiac complications, focusing on SIRT1‐mediated mechanisms.

**Methods:**

Rats (*n* = 8/group) included control, NAR, DCM, DCM + NAR, and DCM + NAR + EX‐527 (SIRT1 inhibitor). After 8 weeks, metabolic profiles, cardiac function, histopathology, oxidative stress (Nuclear Factor Erythroid–Related Factor 2 [Nrf2], MDA, SOD, and GSH), inflammation (nuclear factor kappa B [NF‐*κ*B], TNF‐*α*, and IL‐6), apoptosis (Bax/Bcl‐2 and Caspase‐3), fibrosis (transforming growth factor beta [TGF‐*β*] and Smad2), and SIRT1 signaling were analyzed.

**Results:**

NAR exhibited hypoglycemic effects, stimulated the release of insulin, and enhanced the lipid profile, thus maintaining the structural integrity of cardiomyocytes and preventing an increase in heart weight, while also restoring the histomorphology in DCM rats. Moreover, NAR influenced both systolic and diastolic blood pressure; reduced serum levels of CK‐MB, BNP, and troponin T; downregulated the activity of TGF‐*β*, Smad2, and Caspase‐3; and decreased the Bax/Bcl‐2 ratio. Crucially, NAR upregulated SIRT1 and modulated the levels of Nrf2, NF‐*κ*B, Smad2, and Forkhead Box Protein O1 (FOXO1) in the cardiac tissues of the DCM group. These findings were associated with a significant reduction in oxidative stress and inflammation, alongside diminished NF‐*κ*B activity, while promoting Nrf2 activity and Bcl‐2. Importantly, the effects of NAR were counteracted by EX‐527.

**Conclusion:**

NAR protects against DCM primarily through SIRT1 activation, integrating hypoglycemic, antioxidant, anti‐inflammatory, antifibrotic, and antiapoptotic actions. SIRT1 represents a promising target for flavonoid‐based DCM therapy.

## 1. Introduction

Diabetes mellitus has emerged as a critical global health challenge, with projections estimating that over 643 million individuals worldwide will be affected by 2030 [1]. Among cardiovascular diseases (CVDs), diabetic cardiomyopathy (DCM) is a distinct clinical entity characterized by ventricular dysfunction that arises independently of coronary artery disease or hypertension [2]. DCM arises from sustained hyperglycemia, insulin resistance, and dyslipidemia, which collectively impair metabolic flexibility in cardiomyocytes [3]. Heightened oxidation of glucose and fatty acids and imbalanced levels of reactive oxygen species (ROS) disrupt cellular calcium balance, promote lipid peroxidation, and trigger nuclear factor kappa B (NF‐*κ*B) signaling that upregulates proinflammatory cytokines. Furthermore, reduced activity of Nuclear Factor Erythroid 2–Related Factor 2 (Nrf2) impairs the antioxidant response, exacerbating oxidative damage and inflammatory injury. Despite advancements in understanding DCM and improving glycemic control, the condition remains underdiagnosed and inadequately treated. Its asymptomatic early stages pose significant diagnostic challenges, whereas current treatments fail to effectively target the underlying molecular mechanisms. This highlights the urgent need for the development of therapies that can prevent or reverse diabetic cardiac dysfunction [4].

The NAD‐dependent deacetylase named Silent Information Regulator 1 (SIRT1) has emerged as a key regulator of cellular responses to metabolic and oxidative stress. In experimental models of DCM, reduced SIRT1 expression and activity contribute to increased oxidative damage, inflammation, and mitochondrial dysfunction [5]. Activating SIRT1, either through genetic overexpression or compounds such as resveratrol, has been demonstrated to counteract these detrimental effects, enhancing overall cardiac performance. Emerging preclinical studies highlight the cardioprotective effects of SIRT1 in metabolic disorders, positioning it as a promising therapeutic target for mitigating diabetic cardiovascular complications [6, 7]. However, its interaction with flavonoid‐based therapies in DCM remains insufficiently studied.

Naringenin (NAR), the aglycone form of the citrus flavonoid naringin, exhibits potent antidiabetic, antioxidant, anti‐inflammatory, and antifibrotic properties, making it a promising candidate for various pharmacological applications. A number of investigations have previously explored the cardioprotective properties of NAR in diabetic models. He et al. illustrated that NAR mitigates myocardial damage in streptozotocin (STZ)‐induced diabetic mice by modulating the Nrf2 and NF‐*κ*B signaling pathways [8]. Li et al. indicated that NAR reduces myocardial ischemia/reperfusion injury in STZ‐induced diabetic rats through the enhancement of the miR‐126‐PI3K/AKT axis [9]. Zhang et al. demonstrated that NAR inhibits cardiomyocyte hypertrophy via the activation of EETs/PPARs [10], whereas Xu et al. offered a thorough review of the oxidative stress signaling pathways implicated in DCM and the prospective therapeutic role of NAR [11]. Nevertheless, despite these significant findings, the involvement of SIRT1 signaling in mediating the cardioprotective effects of NAR against DCM remains unexplored. Additionally, no research has utilized a SIRT1‐specific inhibitor to conclusively determine the causal link between SIRT1 activation and the multitargeted effects of NAR. Therefore, the present study sought to investigate the role of NAR in the STZ‐induced rat model of DCM, concentrating on SIRT1‐mediated pathways, thus addressing this essential knowledge gap.

Collectively, SIRT1 is a master regulator of cellular responses to metabolic and oxidative stress, with established roles in improving insulin sensitivity, reducing oxidative damage, and suppressing inflammation, all of which are central to diabetic cardiac pathology. Given that NAR′s cardioprotective effects have been linked to multiple pathways but never to SIRT1, and given that SIRT1 activation represents a promising therapeutic strategy for diabetic complications, we believe this investigation is both mechanistically novel and clinically relevant. By exploring this novel mechanism, the study moves beyond glucose‐centric approaches, potentially advancing flavonoid‐based, multitargeted therapies for diabetic cardiovascular complications.

## 2. Materials and Methods

### 2.1. Experimental Subjects and Study Design

This investigation utilized a validated STZ‐induced DCM model in adult male Wistar rats to assess the cardioprotective potential of NAR and explore the role of SIRT1 signaling. Although the STZ‐induced diabetic rat model does not perfectly replicate all clinical criteria of DCM, particularly the absence of hypertension as an exclusion criterion, it remains one of the most extensively validated preclinical models for studying diabetes‐associated cardiac pathology, encompassing key features such as myocardial fibrosis, oxidative stress, inflammation, and apoptosis [12–14]. Adult male Wistar rats (200–250 g, 8–10 weeks old) were maintained under controlled environmental conditions (22^°^C ± 2^°^C, 12‐h light/dark cycle) with ad libitum access to standard feed and water. Following a 7‐day acclimatization period (Days 0–6), the rats were randomly divided into five experimental cohorts (*n* = 8 per group) including control (CON) (untreated healthy rats), NAR (healthy rats administered NAR), DCM (STZ‐induced diabetic rats), DCM + NAR (diabetic rats treated with NAR), and DCM + NAR + EX‐527 (diabetic rats receiving NAR combined with EX‐527, a selective SIRT1 inhibitor).

Diabetes was induced in rats by a single intraperitoneal (i.p.) injection of STZ (60 mg/kg, Ab142155, Abcam, Cambridge, United Kingdom) dissolved in citrate buffer (pH 4.5) after an overnight fast. The STZ injection was administered on Day 7 of the experimental protocol, immediately following the 7‐day acclimatization period. To avoid acute hypoglycemia, animals received a 5% glucose solution in drinking water for 24 h after injection. CON rats received an equal volume of citrate buffer without STZ. Diabetes was confirmed by measuring fasting blood glucose levels 72 h after injection (Day 10), followed by assessments on three consecutive days (Days 10, 11, and 12). Only rats with sustained hyperglycemia (> 250 mg/dL) over all three consecutive assessments were included in the study. Nondiabetic CON rats maintained normoglycemia, with fasting glucose levels ranging between 80 and 100 mg/dL.

NAR (Sigma‐Aldrich, purity ≥ 95%) was administered daily via oral gavage at a dose of 50 mg/kg body weight for 8 weeks, commencing 1 week after STZ injection (Day 14). This dosage was selected based on previous studies demonstrating optimal cardioprotective efficacy in STZ‐induced diabetic rat models. EX‐527, a selective SIRT1 inhibitor, was delivered intraperitoneally (5 mg/kg) every 48 h, 30 min prior to NAR administration, following a validated in vivo protocol for SIRT1 inhibition [15]. CON cohorts received vehicle solutions matched to each treatment (0.5% carboxymethylcellulose for NAR and saline for EX‐527) in equivalent volumes. Body weight was monitored at biweekly intervals (Days 14, 28, 42, 56, and 70) over the 56‐day experimental period (Figure 1).

**Figure 1:**
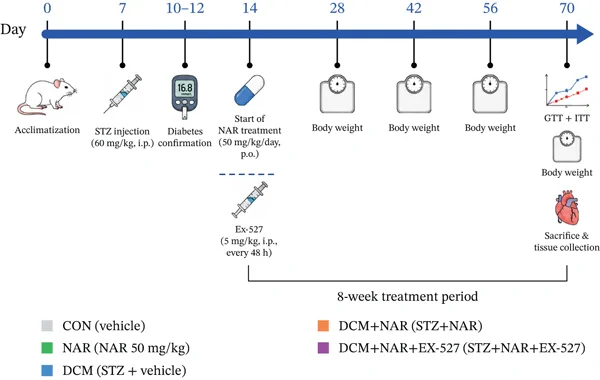
Experimental timeline. Schematic representation of the 70‐day experimental protocol. Day 0: start of 7‐day acclimatization period. Day 7: single intraperitoneal injection of streptozotocin (STZ) (60 mg/kg) to induce diabetes. Days 10–12: diabetes confirmation based on fasting blood glucose (FBG) > 250 mg/dL over three consecutive days. Day 14: commencement of naringenin (NAR) (50 mg/kg/day, oral gavage) treatment for the NAR, DCM + NAR, and DCM + NAR + EX‐527 groups; EX‐527 (5 mg/kg, i.p., every 48 h) coadministration for the DCM + NAR + EX‐527 group. Body weight was monitored at biweekly intervals on Days 14, 28, 42, 56, and 70. Day 70 (Week 8 of treatment): glucose tolerance test (GTT) and insulin tolerance test (ITT) followed by sacrifice and tissue collection. CON, control; DCM, diabetic cardiomyopathy; CMC, carboxymethylcellulose; p.o., per os (oral); i.p., intraperitoneal.

### 2.2. Noninvasive Measurement of Blood Pressure

Noninvasive measurements of systolic blood pressure (SBP) and diastolic blood pressure (DBP) were conducted in conscious rats using the CODA High‐Throughput Tail‐Cuff System (Kent Scientific, Torrington, Connecticut, United States). A 3‐day acclimatization protocol for restraint procedures and the warming platform was implemented for all animals before baseline data collection. Weekly blood pressure recordings were performed between 9:00 and 11:00 AM, with each session consisting of 10 acclimation cycles followed by eight valid measurement cycles per subject. SBP and DBP values were automatically calculated by the system′s volume–pressure recording (VPR) sensors and averaged across successful measurement cycles. Data were excluded from analysis if intrasession variability exceeded 20 mmHg.

### 2.3. The Glucose Tolerance Test (GTT) and Insulin Tolerance Test

Following an 8‐week treatment period, glucose and insulin tolerance assessments were conducted in rats using standardized procedures. Before the GTT, rats underwent a 12‐h overnight fasting period to establish baseline metabolic conditions. Blood samples were initially collected from the caudal vein using an Ultra Touch glucometer (Trividia Health, Inc.) to measure fasting glucose levels. Subsequently, a glucose solution (2 g/kg body weight) was administered via i.p. injection. Postinjection glucose concentrations were monitored at specified intervals (0, 10, 20, 30, 60, 90, and 120 min) using sterile techniques to extract approximately 2 *μ*L of blood from the caudal vein at each time point. The area under the curve (AUC) for glucose values during the GTT was calculated using the trapezoidal method to provide a comprehensive measure of glucose tolerance. For the ITT, rats fasted for 6 h before receiving an i.p. injection of insulin (1 unit/kg body weight). Blood glucose levels were assessed at 15, 30, 45, and 60 min after administration using the same glucometric methodology. The AUC for glucose values during the ITT was similarly calculated using the trapezoidal method to assess insulin sensitivity. Sterile needles ensured consistent and hygienic blood sampling throughout the procedure. For the GTT, a 12‐h fasting period was employed as this is the standard protocol for assessing glucose tolerance in STZ‐induced diabetic rat models. For the ITT, a 6‐h fasting period was selected based on established protocols for this model. Although shorter fasting durations (2–4 h) have been proposed for ITT to minimize hepatic glycogen depletion and hypoglycemia risk, the 6‐h protocol used in this study is well validated in the literature and employs a relatively low insulin dose (1 U/kg), which has been demonstrated to be safe in diabetic rats [16–18].

### 2.4. Tissue Collection and Processing

Following the therapeutic intervention, the animals were administered a general anesthetic comprising ketamine 80 mg/kg and xylazine 10 mg/kg intraperitoneally. Blood samples (5 mL) were collected via cardiac puncture and allowed to clot at room temperature for 20 min before centrifugation at 2000 RPM. The separated serum was aliquoted and stored at −80°C for subsequent biochemical analyses. Concurrently, cardiac tissues were excised and rinsed thoroughly with cold sterile phosphate‐buffered saline (PBS) before homogenization to remove blood contaminants. Depending on experimental requirements, the tissue samples were either fixed or rapidly snap‐frozen at −80°C to preserve their integrity for further investigation.

### 2.5. Assessment of Metabolic Parameters

Metabolic parameter analysis was conducted through standardized biochemical assessments of blood plasma constituents. Key biomarkers, including creatine kinase‐MB (CK‐MB), B‐type natriuretic peptide (BNP), troponin T, low‐density lipoprotein cholesterol (LDL‐c), high‐density lipoprotein cholesterol (HDL‐c), triglycerides (TGs), total cholesterol (TC), insulin, and glucose, were quantified using commercially available kits (MyBioSource, United States) following the manufacturer′s validated protocols. Insulin resistance was evaluated through the homeostatic model assessment for insulin resistance (HOMA‐IR) index, calculated using fasting glucose and insulin concentrations. The HOMA‐IR value was derived from the formula (fasting glucose [mmol/L] and fasting insulin [*μ*U/mL]):

Insulin resistance=fasting glucose×fasting insulin22.5.

Additionally, pancreatic *β*‐cell function was assessed using the homeostatic model assessment for *β*‐cell function (HOMA‐*β*), calculated as

360×fasting insulin μU/mLFasting glucose mmol/L−63.

### 2.6. Histopathological Examination

The cardiac tissues that were isolated were preserved in buffered formalin (10%) for 1 day, after which dehydration of the tissues was conducted through a graded series of alcohol. Subsequently, the tissues were embedded in paraffin to create sections with a thickness of 5 *μ*m, utilizing a standard microtome (Viabrembo, Milan, Italy). The sections underwent dewaxing with xylene, followed by hydration with ethanol, and ultimately, the sections were rinsed with sterile water and stained with H&E (hematoxylin and eosin). The stained sections were evaluated under a standard microscope (Nikon, Tokyo, Japan).

### 2.7. Enzyme‐Linked Immunosorbent Assay

Cardiac tissue samples from rat models were homogenized in RIPA lysis buffer containing protease inhibitors to prevent protein degradation. A detailed biomarker analysis was then performed using commercial ELISA kits (MyBioSource, United States) following the manufacturer′s instructions. The study quantified fibrotic mediators, including transforming growth factor‐beta (TGF‐*β*) and Smad2; apoptotic regulators such as Caspase‐3 (CASP‐3) activity, Bax, and Bcl‐2; oxidative stress markers like Nrf2, malondialdehyde (MDA), glutathione [19], and superoxide dismutase (SOD) activity; inflammatory mediators, specifically NF‐*κ*B, tumor necrosis factor‐alpha (TNF‐*α*), and Interleukin‐6 (IL‐6); and regulatory proteins including SIRT1 and Forkhead Box Protein O1 (FOXO1). All measurements were conducted in duplicate, with absorbance values normalized to total protein concentration to ensure accuracy and reduce experimental variability.

### 2.8. Gene Expression Analysis

Cardiac tissues collected from rat specimens were homogenized, and total RNA was extracted using TRIzol reagent (Invitrogen, United States) following the manufacturer′s instructions. RNA purity and concentration were assessed spectrophotometrically using a NanoDrop system (BioTek, United States). Subsequently, complementary DNA (cDNA) was synthesized from the isolated RNA using a High‐Capacity cDNA Reverse Transcription Kit (Thermo Fisher Scientific, United States; Cat. #4368814). Quantitative PCR was performed on a StepOne Real‐Time PCR System (Applied Biosystems, United States) with Maxima SYBR Green qPCR Master Mix (Thermo Fisher Scientific, United States; Cat. #K0253). Gene expression levels were normalized to the endogenous control gene *β-actin*, and relative quantification was calculated using the 2^−*Δ* *Δ*CT^ method. The thermal cycling conditions consisted of an initial denaturation step at 95°C for 5 min, followed by 40 cycles of denaturation (95°C, 15 s), annealing (58°C, 15 s), and extension (72°C, 30 s). The primer sequences used for target gene amplification are listed in Table S1. All experiments were performed in triplicate to ensure reproducibility.

### 2.9. Statistical Analysis

All quantitative data are presented as mean ± standard deviation (SD). Statistical comparisons for metabolic parameters, cardiac biomarkers, protein levels, and gene expression were conducted using one‐way ANOVA, followed by Tukey′s post hoc test to evaluate group differences. For GTT and ITT, repeated measures were analyzed using two‐way ANOVA with Sidak′s multiple comparisons test to assess differences at each time point. The AUC for GTT and ITT was calculated using the trapezoidal method with baseline set to zero (*Y* = 0) in GraphPad Prism, and AUC values were compared using one‐way ANOVA with Tukey′s post hoc test. Analyses were performed using GraphPad Prism Version 8 (San Diego, California, United States). A threshold of *p* < 0.05 was applied to determine statistical significance.

## 3. Results

### 3.1. NAR Alleviated Physical, Cardiac, and Metabolic Indices in Rats With DCM

As Figure 2A depicts, the weight of the DCM and DCM + NAR + EX‐527 groups significantly decreased during the treatment periods (*p* < 0.001). However, the DCM + NAR group showed significant differences compared with both the CON and DCM groups (*p* < 0.05). Conversely, the assessment of cardiac hypertrophy by the measurement of heart‐to‐body weight (H/B) ratio revealed that DCM + NAR caused a significant reduction compared with DCM (*p* < 0.001) and DCM + NAR + EX‐527 animals (*p* = 0.004), although it had no significant difference compared with the CON group (*p* = 0.082, Figure 2B). Furthermore, SBP (Figure 2C) and DBP (Figure 2D) in DCM and DCM + NAR + EX‐527 were significantly higher than in CON animals (*p* < 0.001), whereas treatment of DCM animals with NAR caused a significant reduction in both SBP and DBP levels compared with the DCM group (*p* < 0.01).

**Figure 2:**
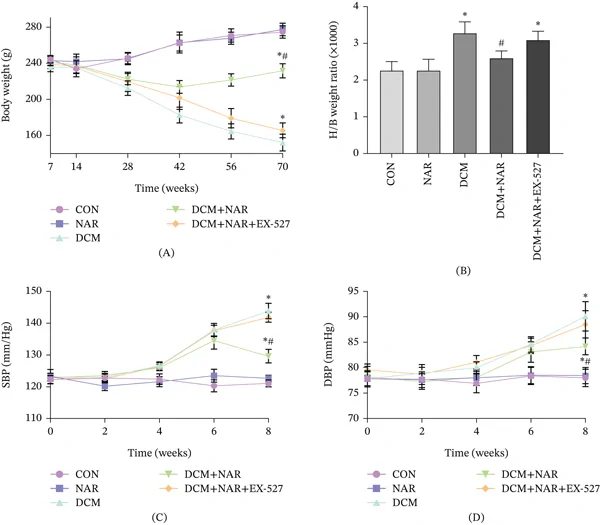
NAR improves physiological and metabolic parameters in STZ‐induced DCM rats. (A) Total body weight, (B) heart‐to‐body (H/B) weight ratio, (C) systolic blood pressure (SBP), and (D) diastolic blood pressure (DBP). Data = mean ± SD; *n* = 8 rats/group. Statistical analysis was performed using one‐way ANOVA with Tukey′s post hoc test. Groups: CON (untreated control), NAR (naringenin‐only), DCM (STZ‐induced diabetic cardiomyopathy), DCM + NAR (NAR‐treated diabetic), and DCM + NAR + EX‐527 (diabetic animals treated with NAR + SIRT1 inhibitor).  ^∗^ *p* < 0.05 vs. CON, ^#^ *p* < 0.05 vs. DCM, and  ^∗^^#^ *p* < 0.05 vs. both CON and DCM; ns, not significant.

The GTT was performed to evaluate glucose tolerance following an i.p. glucose load (Figure 3A). Two‐way repeated‐measures ANOVA revealed significant main effects of group (*F*(4, 35) = 127.2, *p* < 0.0001), time (*F*(6,210) = 18.8, *p* < 0.0001), and their interaction (*F*(24,210) = 3.11, *p* < 0.0001). The DCM group exhibited significantly elevated glucose levels compared with the CON group at all postglucose time points (*p* < 0.0001), confirming severe glucose intolerance. NAR treatment significantly reduced glucose levels in diabetic rats compared with the DCM group at all time points (*p* < 0.0001), though glucose levels remained partially elevated compared with CON (*p* < 0.01 at 10, 20, 60, and 120 min). Coadministration of EX‐527 with NAR completely abolished these effects, as the DCM + NAR + EX‐527 group showed no significant difference from the DCM group at any time point (*p* > 0.05). NAR alone had no effect on glucose tolerance in healthy rats (*p* > 0.05 vs. CON at all time points). AUC analysis of the GTT revealed a significant overall difference among groups (*F*(4, 35) = 727.2, *p* < 0.0001, Figure 3B). The DCM group exhibited a markedly elevated AUC compared with the CON group (33,884 ± 290.7 vs. 18,662 ± 167.3, *p* < 0.0001). NAR treatment significantly reduced the AUC in diabetic rats (21,879 ± 280.2, *p* < 0.0001 vs. DCM), whereas EX‐527 cotreatment abolished this protective effect (33,180 ± 394.8, *p* < 0.0001 vs. DCM + NAR; *p* > 0.05 vs. DCM). NAR alone had no significant effect on AUC in healthy rats (18,444 ± 168.6, *p* > 0.05 vs. CON). The ITT was performed to assess insulin sensitivity following an i.p. insulin injection (Figure 3C). Two‐way repeated‐measures ANOVA revealed significant main effects of group (*F*(4, 35) = 156.8, *p* < 0.0001), time (*F*(4,140) = 42.3, *p* < 0.0001), and their interaction (*F*(16,140) = 8.74, *p* < 0.0001). The DCM group exhibited significantly impaired insulin sensitivity, with higher glucose levels at all postinjection time points compared with the CON group (*p* < 0.0001). NAR treatment significantly improved insulin sensitivity in diabetic rats, as evidenced by lower glucose levels compared with the DCM group at all time points (*p* < 0.001). Coadministration of EX‐527 with NAR completely abolished these beneficial effects, with the DCM + NAR + EX‐527 group showing no significant difference from the DCM group at any time point (*p* > 0.05). NAR alone had no effect on insulin sensitivity in healthy rats (*p* > 0.05 vs. CON at all time points). AUC analysis of the ITT revealed a significant overall difference among groups (*F*(4, 35) = 483.2, *p* < 0.0001, Figure 3D). The DCM group exhibited a markedly elevated AUC compared with the CON group (8558 ± 123.6 vs. 4930 ± 65.54, *p* < 0.0001), confirming severe insulin resistance. NAR treatment significantly reduced the AUC in diabetic rats (6613 ± 103.8, *p* < 0.0001 vs. DCM), whereas EX‐527 cotreatment completely abolished this protective effect (8746 ± 140.0, *p* < 0.0001 vs. DCM + NAR; *p* > 0.05 vs. DCM). NAR alone had no significant effect on AUC in healthy rats (5116 ± 85.98, *p* > 0.05 vs. CON). These findings demonstrate that NAR improves insulin sensitivity in STZ‐induced diabetic rats through a SIRT1‐dependent mechanism.

**Figure 3:**
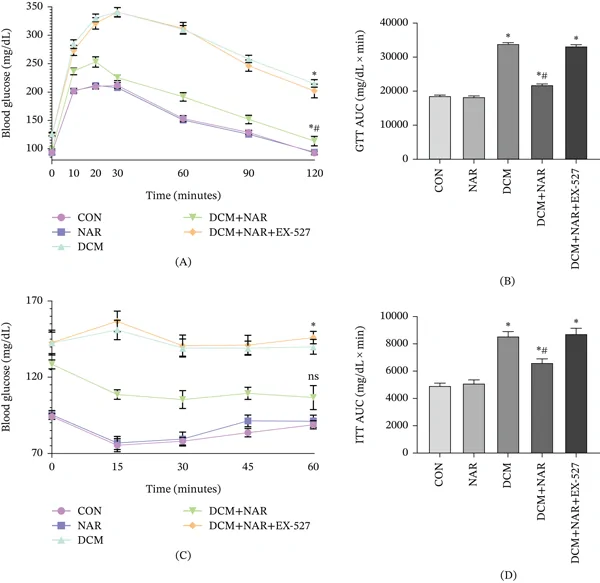
NAR improved glucose and insulin tolerance in diabetic animals. (A) Glucose tolerance test (GTT), (B) area under the curve (AUC) analysis of the GTT, (C) insulin tolerance test (ITT), and (D) AUC analysis of the ITT. AUC was calculated using the trapezoidal method with baseline set to zero (*Y* = 0). Data are presented as mean ± SD; *n* = 8 rats/group. (A,C) GTT and ITT curves were analyzed using two‐way repeated‐measures ANOVA with Sidak′s multiple comparisons test. (B, D) AUC values were analyzed using one‐way ANOVA with Tukey′s post hoc test.  ^∗^ *p* < 0.05 vs. CON, ^#^ *p* < 0.05 vs. DCM, and  ^∗^^#^ *p* < 0.05 vs. both CON and DCM; ns, not significant.

HOMA‐IR, a marker of insulin resistance, was significantly elevated in the DCM group compared with the CON group (9.605 ± 0.483 vs. 4.596 ± 0.117, *p* < 0.0001), indicating severe insulin resistance. NAR treatment significantly reduced HOMA‐IR in diabetic rats (6.125 ± 0.475, *p* < 0.0001 vs. DCM), although values remained significantly elevated compared with CON (*p* = 0.0015). Coadministration of EX‐527 with NAR completely abolished this protective effect, as the DCM + NAR + EX‐527 group showed no significant difference from the DCM group (9.762 ± 0.339, *p* > 0.05 vs. DCM) and a significant increase compared with the DCM + NAR group (*p* < 0.0001). NAR alone had no significant effect on HOMA‐IR in healthy rats (4.617 ± 0.151, *p* > 0.05 vs. CON). To evaluate pancreatic *β*‐cell function, HOMA‐*β* was calculated using fasting glucose and insulin levels. One‐way ANOVA revealed a significant overall difference among groups (*F*(4, 35) = 142.6, *p* < 0.0001). The DCM group exhibited severely impaired *β*‐cell function compared with the CON group (22.72 ± 1.21 vs. 262.30 ± 29.10, *p* < 0.0001), confirming STZ‐induced pancreatic *β*‐cell destruction. NAR treatment significantly improved *β*‐cell function in diabetic rats (85.04 ± 7.47, *p* < 0.0001 vs. DCM), although HOMA‐*β* remained significantly lower than CON (*p* < 0.0001). Coadministration of EX‐527 with NAR completely abolished this protective effect, as the DCM + NAR + EX‐527 group showed no significant difference from the DCM group (27.12 ± 1.38, *p* > 0.05 vs. DCM). NAR alone had no significant effect on HOMA‐*β* in healthy rats (278.57 ± 30.31, *p* > 0.05 vs. CON) (Table 1).

**Table 1:** NAR alleviated glycemic, lipid, and cardiac profiles in rats with DCM.

Parameter	CON	NAR	DCM	DCM + NAR	DCM + NAR + EX‐527
Insulin (mU/L)	20.24 ± 1.69	20.61 ± 1.91	13.74 ± 1.47^∗^	17.62 ± 2.92^∗^^#^	15.00 ± 1.54^∗^
Insulin resistance	4.60 ± 0.33	4.62 ± 0.43	9.61 ± 1.37^∗^	6.13 ± 1.34	9.76 ± 0.96^∗^
HOMA‐*β*	262.30 ± 29.10	278.57 ± 30.31	22.72 ± 1.21^∗^	85.04 ± 7.47^∗^^#^	27.12 ± 1.38^∗^
Glucose (mg/dL)	92.16 ± 5.31	90.81 ± 4.75	282.9 ± 26.57^∗^	140.59 ± 16.69^∗^^#^	264.02 ± 18.50^∗^
TG (mg/dL)	47.93 ± 2.67	45.66 ± 4.94	228.92 ± 13.13^∗^	66.97 ± 6.11^∗^^#^	206.20 ± 10.97^∗^
TC (mg/dL)	65.59 ± 5.48	61.26 ± 6.64	136.52 ± 14.07^∗^	103.59 ± 16.46^∗^^#^	120.89 ± 9.35^∗^
LDL (mg/dL)	23.82 ± 2.50	22.70 ± 2.33	91.24 ± 3.04^∗^	39.51 ± 6.57^∗^^#^	90.28 ± 4.34^∗^
HDL (mg/dL)	36.42 ± 1.95	40.73 ± 1.89	24.61 ± 1.92^∗^	33.65 ± 4.93	28.38 ± 4.26^∗^
CK‐MB (U/L)	103.71 ± 9.93	106.85 ± 8.97	307.57 ± 17.01^∗^	144.21 ± 29.66^∗^^#^	290.28 ± 32.30^∗^
BNP (ng/mL)	1.11 ± 0.36	0.99 ± 0.22	3.86 ± 0.30^∗^	2.09 ± 0.68^∗^^#^	3.46 ± 0.49^∗^
Troponin T (ng/L)	2.95 ± 0.61	2.78 ± 0.57	161.06 ± 12.22^∗^	49.58 ± 8.25^∗^^#^	148.49 ± 12.49^∗^

*Note:* Data presented as *m* *e* *a* *n* ± *S* *D*; *n* = 8 rats/group.

^∗^ *p* < 0.05 vs. CON, ^#^ *p* < 0.05 vs. DCM, and  ^∗^^#^ *p* < 0.05 vs. both CON and DCM.

Moreover, the levels of TC, TG, and LDL significantly increased in the DCM and DCM + NAR + EX‐527 groups compared with CON, whereas HDL levels showed a significant decrease when compared with CON (*p* < 0.001). Importantly, the treatment of DCM animals with NAR significantly restored the levels of glycemic and lipid profile in comparison with the DCM group (*p* < 0.01). Along with that, the assessment of cardiac indices, including CK‐MB, BNP, and troponin T, revealed a significant increment in the levels of all three measured factors in DCM animals compared with the CON group (*p* < 0.001). Although treatment with NAR in DCM animals significantly reduced the levels of CK‐MB, BNP, and troponin T compared with DCM animals (*p* < 0.001), no difference between DCM and DCM + NAR + EX‐527 was achieved (*p* > 0.05). Importantly, the NAR and CON groups showed no significant differences regarding the mentioned physical, cardiac, and metabolic indices (*p* > 0.05).

### 3.2. The Histomorphology of Cardiac Tissue in DCM Rats Was Improved by NAR Treatment

The analysis of the histomorphology of cardiac tissue of CON and NAR animals showed striated cardiomyocytes containing nuclei located in the center of cells and anastomosed/branched cell fibers arranged in linear arrays. Meanwhile, the cardiomyocytes′ striations and anastomoses were mainly lost, and discs were intercalated in both the DCM and DCM + NAR + EX‐527 groups. Notably, DCM + NAR animals showed qualitatively improved histoarchitecture, characterized by better‐preserved striations and reduced interstitial edema, compared with DCM animals. Histopathological evaluation was performed by two independent observers blinded to the experimental groups (Figure 4).

**Figure 4:**
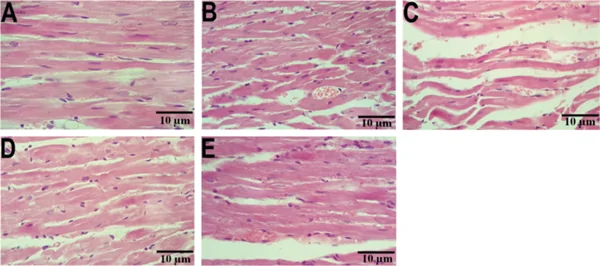
NAR preserves cardiac histoarchitecture in DCM. Representative H&E‐stained myocardial sections (scale bar: 10 *μ*m). Histopathological assessment was performed qualitatively by two independent observers blinded to the experimental groups. (A) CON: organized cardiomyocytes with central nuclei and striations. (B) NAR: normal architecture. (C) DCM: disrupted striations, interstitial edema, and nuclear pyknosis. (D) DCM + NAR: restored striations and reduced edema. (E) DCM + NAR + EX‐527: pathology similar to DCM.

### 3.3. NAR Protects Cardiac Tissue From DCM‐Induced Apoptosis and Fibrosis

The present findings revealed that cardiac tissue of DCM animals exhibited enhanced apoptosis, characterized by significantly increased *Bax* expression (*p* = 0.001, Figure 5B) and *CASP-3* expression (*p* < 0.001, Figure 5C), alongside significantly decreased *Bcl-2* expression (*p* < 0.001, Figure 5A), compared with CON. Consistent with gene expression findings, protein levels of Bax (Figure 5F) and CASP‐3 (Figure 5G) activity were significantly increased, whereas Bcl‐2 (Figure 5E) protein levels were significantly decreased, in the cardiac tissue of DCM animals compared with CON (*p* < 0.001). Notably, NAR treatment significantly increased Bcl‐2 expression (both gene and protein) and decreased Bax and CASP‐3 expression (both gene and protein) in the cardiac tissue of diabetic rats compared with the DCM group (*p* < 0.01 for Bax and CASP‐3; *p* < 0.001 for Bcl‐2) (Figure 5). Furthermore, NAR treatment significantly reduced the elevated levels of TGF‐*β* (Figure 6A), Smad2 (Figure 6B), and the Bax/Bcl‐2 ratio (Figure 5D) in DCM rats (*p* < 0.01). No significant differences were observed between the DCM and DCM + NAR + EX‐527 groups, nor between the CON and NAR groups, for any of the measured apoptotic and fibrotic markers (*p* > 0.05, Figure 6).

**Figure 5:**
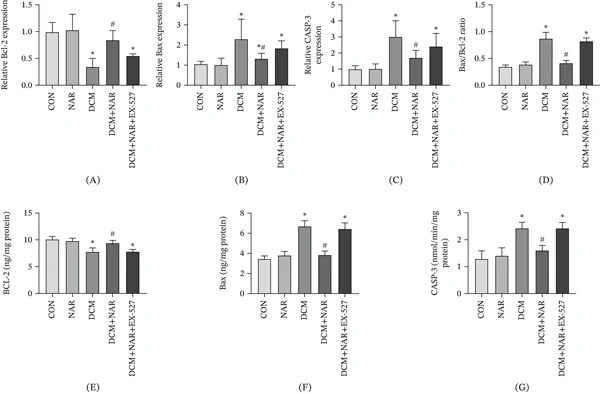
NAR attenuates cardiac apoptosis and fibrosis. (A–C) mRNA expression of *Bcl-2*, *Bax*, and *CASP-3*. (D–F) Protein levels/activity. (G) Bax/Bcl‐2 ratio. Data = mean ± SD; *n* = 8. Statistical analysis was performed using one‐way ANOVA with Tukey′s post hoc test.  ^∗^ *p* < 0.05 vs. CON, ^#^ *p* < 0.05 vs. DCM, and  ^∗^^#^ *p* < 0.05 vs. both CON and DCM.

**Figure 6:**
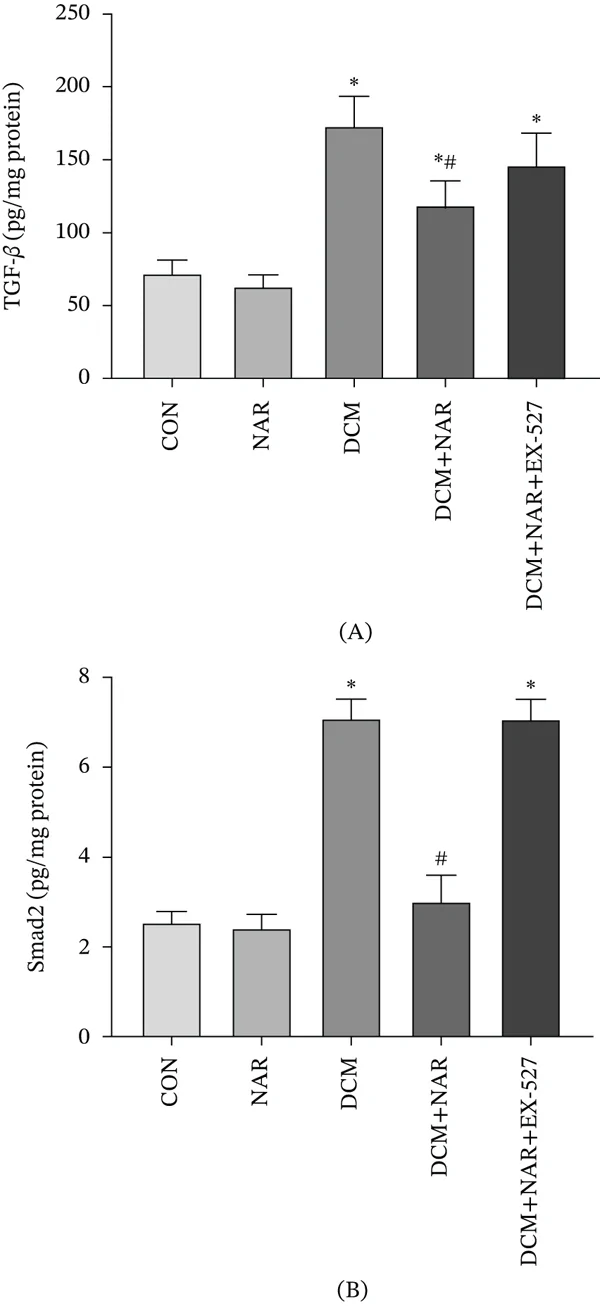
NAR modulated markers of fibrosis in the cardiac tissue of diabetic animals. (A) TGF‐*β* levels and (B) Smad2 levels. Data = mean ± SD; *n* = 8. Statistical analysis was performed using one‐way ANOVA with Tukey′s post hoc test.  ^∗^ *p* < 0.05 vs. CON, ^#^ *p* < 0.05 vs. DCM, and  ^∗^^#^ *p* < 0.05 vs. both CON and DCM.

### 3.4. NAR Suppressed Inflammation and Oxidative Stress in the Cardiac Tissue of Animals With DCM

The assessment of inflammation was performed by measuring NF‐*κ*B mRNA expression (qRT‐PCR) and protein levels (ELISA), along with protein levels of TNF‐*α* and IL‐6 (ELISA) in cardiac tissue. Findings revealed that in the cardiac tissue of DCM and DCM + NAR + EX‐527 animals, NF‐*κ*B gene expression and protein levels (Figure 7A,B, respectively) were significantly increased compared with the CON group (*p* < 0.001). Similarly, protein levels of TNF‐*α* (Figure 7C) and IL‐6 (Figure 7D) were significantly elevated in the DCM and DCM + NAR + EX‐527 groups compared with CON (*p* < 0.001). NAR treatment significantly reduced NF‐*κ*B protein levels in diabetic rats compared with the DCM group (*p* < 0.001); however, NF‐*κ*B gene expression in the DCM + NAR group remained significantly elevated compared with both the CON (*p* = 0.005) and DCM (*p* < 0.001) groups. Additionally, NAR significantly reduced TNF‐*α* and IL‐6 protein levels in cardiac tissue of diabetic rats (*p* < 0.001 vs. DCM).

**Figure 7:**
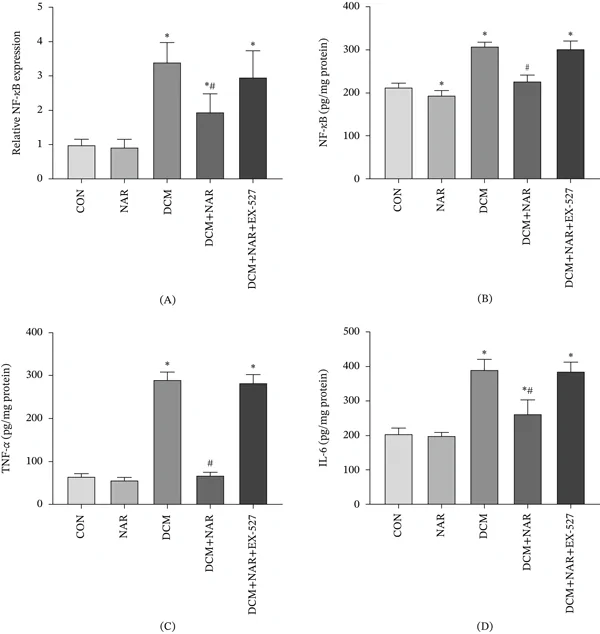
NAR suppresses cardiac inflammatory mediators in diabetic hearts. (A,B) NF‐*κ*B expression (mRNA/protein), (C) TNF‐*α* levels, and (D) IL‐6 levels. Statistical analysis was performed using one‐way ANOVA with Tukey′s post hoc test. Data = mean ± SD; *n* = 8.  ^∗^ *p* < 0.05 vs. CON, ^#^ *p* < 0.05 vs. DCM, and  ^∗^^#^ *p* < 0.05 vs. both CON and DCM; (*p* < 0.05).

Nrf2 gene expression and protein levels (Figure 8A,B, respectively) were significantly suppressed in DCM animals compared with CON (*p* < 0.001). NAR treatment significantly increased both Nrf2 gene expression and protein levels compared with DCM (*p* < 0.01). However, despite this improvement, *Nrf2* mRNA levels in the DCM + NAR group remained significantly lower than CON (*p* = 0.002). Moreover, GSH levels (Figure 8D) and SOD activity (Figure 8C) were significantly reduced in DCM animals compared with CON (*p* < 0.001), whereas NAR treatment significantly increased both markers (*p* < 0.01 vs. DCM). MDA levels (Figure 8E) were markedly elevated in DCM animals compared with CON (*p* < 0.001), and NAR treatment significantly reduced MDA levels compared with both CON (*p* = 0.042) and DCM (*p* < 0.001). Notably, no significant differences were observed between the DCM and DCM + NAR + EX‐527 groups for any of the measured parameters (*p* > 0.05).

**Figure 8:**
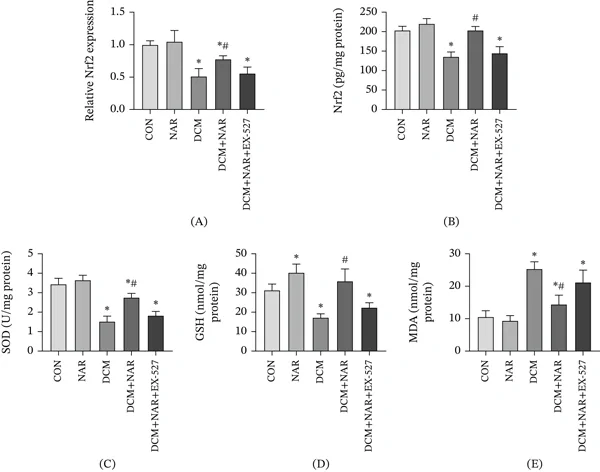
NAR alleviates oxidative stress in diabetic hearts. (A,B) Nrf2 expression (mRNA/protein), (C) SOD activity, (D) GSH levels, and (E) MDA levels. Data = mean ± SD; *n* = 8. Statistical analysis was performed using one‐way ANOVA with Tukey′s post hoc test.  ^∗^ *p* < 0.05 vs. CON, ^#^ *p* < 0.05 vs. DCM, and  ^∗^^#^ *p* < 0.05 vs. both CON and DCM; (*p* < 0.05).

### 3.5. NAR Restored the Expression of Upstream Regulators in the Cardiac Tissue of DCM Animals

To determine whether NAR′s protective effects are mediated through SIRT1 signaling, we assessed SIRT1 and FOXO1 expression at both mRNA (qRT‐PCR) and protein (ELISA) levels. In DCM animals, *SIRT1* gene expression (Figure 9A) and protein (Figure 9C) levels were significantly reduced, whereas *FOXO1* expression (Figure 9B) was significantly elevated, compared with the CON group (*p* < 0.001). NAR treatment significantly increased *SIRT1* expression (*p* = 0.001) and decreased *FOXO1* expression (*p* < 0.001) compared with the DCM group. However, *FOXO1* expression in the DCM + NAR group remained significantly elevated compared with CON (*p* < 0.001). A similar trend was observed for SIRT1 and FOXO1 protein levels (Figure 9D). Importantly, coadministration of EX‐527 with NAR significantly reversed these effects, as *SIRT1* expression was decreased and FOXO1 expression was increased in the DCM + NAR + EX‐527 group compared with the DCM + NAR group (*p* < 0.01). No significant differences were observed between the DCM and DCM + NAR + EX‐527 groups for either SIRT1 or FOXO1 expression at the mRNA or protein level (*p* > 0.05) (Figure 9).

**Figure 9:**
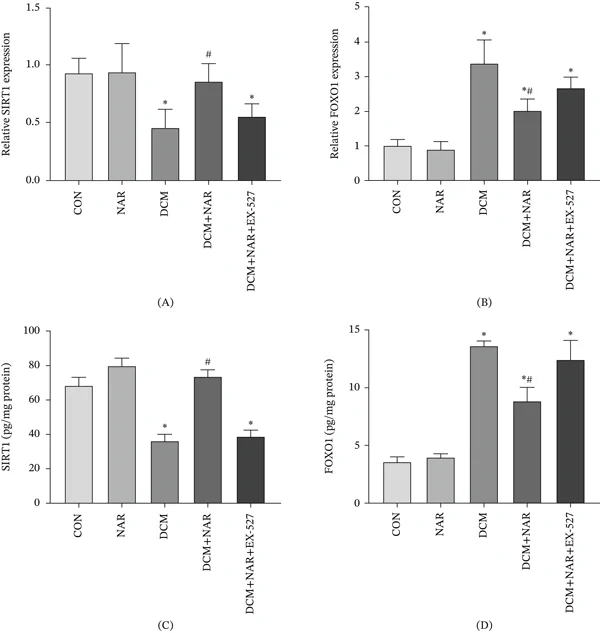
NAR restores SIRT1‐FOXO1 signaling in DCM. (A–D) SIRT1 and FOXO1 expression (mRNA/protein). Data = mean ± SD; *n* = 8. Statistical analysis was performed using one‐way ANOVA with Tukey′s post hoc test.  ^∗^ *p* < 0.05 vs. CON, ^#^ *p* < 0.05 vs. DCM, and  ^∗^^#^ *p* < 0.05 vs. both CON and DCM (*p* < 0.05).

## 4. Discussion

Limited studies have explored the cardioprotective actions of NAR in experimental animal models of DCM, particularly in those representing T1DM. The single high‐dose STZ injection (60 mg/kg) used in this study induces severe pancreatic *β*‐cell destruction, resulting in an insulin‐deficient state with marked hyperglycemia, weight loss, and hypoinsulinemia, features consistent with T1DM [20]. This contrasts with T2DM models, which typically require a high‐fat diet combined with low‐dose STZ to induce insulin resistance with hyperinsulinemia [20]. Distinct patterns of cardiac dysfunction exist between T1DM and T2DM models, with T1DM showing more pronounced systolic dysfunction, oxidative stress, and fibrosis [21]. Although previous investigations have demonstrated NAR′s cardioprotective effects through Nrf2/NF‐*κ*B regulation, miR‐126‐PI3K/AKT axis activation, and EET/PPAR‐mediated antihypertrophic effects, the involvement of SIRT1 signaling has remained unexplored [8, 9, 11].

In this research, the presence of diabetes complications (DC) in the animal model was confirmed by the end of Week 8, evidenced by significant hyperglycemia, hypoinsulinemia, compromised systolic and diastolic functions, elevated cardiac injury and fibrosis markers, and disrupted biochemical biomarkers. Although NAR has been shown to lower both SBP and DBP in diabetic rats, which may play a role in its cardioprotective properties, multiple lines of evidence suggest a direct mechanism mediated by SIRT1 that is independent of hemodynamic alterations. The overexpression of *SIRT1* in vascular smooth muscle cells leads to a decrease in SBP and inhibits vascular remodeling induced by Angiotensin II, indicating that SIRT1 is directly involved in the regulation of blood pressure [22]. Moreover, SIRT1 mitigates inflammatory responses by deacetylating the NF‐*κ*B p65 subunit, which reduces its transcriptional activity, and bolsters antioxidant defenses through the activation of Nrf2 [23–25]. NAR has been recognized as a natural activator of SIRT1, and its chronic administration increases SIRT1 expression while decreasing the production of ROS in myocardial tissue [26]. Additionally, NAR improves vascular senescence and atherosclerosis via SIRT1‐mediated signaling pathways [27]. Furthermore, the hypoglycemic and insulin‐sensitizing effects of NAR, which were also inhibited by EX‐527, may enhance cardioprotection through metabolic improvements that are independent of changes in blood pressure. A prior investigation indicated that NAR at 50 mg/kg did not result in a reduction of blood pressure in a fructose–STZ diabetic rat model when it was administered 4 weeks after diabetes induction [28]. This inconsistency with present results may be attributed to variations in the timing of the intervention and the type of animal model used. Importantly, although there was no observed improvement in blood pressure, Zaidun et al. noted significant enhancements in oxidative stress and aortic histology [28], which reinforces our assertion that the vascular protective effects of NAR are not contingent upon hemodynamic alterations but are instead mediated through SIRT1.

DCM progresses temporally from early diastolic dysfunction to systolic dysfunction and heart failure. Early stages are marked by left ventricular hypertrophy and diastolic dysfunction, which may later evolve into systolic dysfunction and overt heart failure. In the STZ‐induced diabetic rat model, echocardiographic studies have demonstrated that bradycardia precedes contractile dysfunction, with overt left ventricular systolic and diastolic dysfunction becoming fully manifested after 6 weeks of diabetes [29]. The transition from diastolic to systolic dysfunction is associated with progressive myocardial fibrosis, collagen deposition, and adverse ventricular remodeling. These hallmark features, including diastolic impairment, systolic dysfunction, and myocardial fibrosis, were all observed in the present STZ‐induced diabetic rats and were significantly attenuated by NAR treatment.

Previous studies have documented impaired cardiac function and fibrosis in STZ‐induced diabetic rats at various time points, with fibrosis peaking by Week 8 [30–33]. However, final body weights and heart weights are generally diminished in STZ‐diabetic rats, whereas the H/B ratio is elevated, indicating hypertrophy [20]. Additionally, increased circulating levels of CK‐MB, troponin T, and BNP, well‐established markers for cardiac injury and hypertrophy, were noted in STZ‐diabetic hearts [34, 35]. Hyperglycemia‐induced ROS generation drives inflammation, fibrosis, and apoptosis in diabetic hearts [12, 36, 37]. In fact, ROS can provoke cardiac inflammation by activating NF‐*κ*B. Furthermore, both ROS and inflammatory cytokines contribute to cardiac fibrosis by activating the TGF‐*β*1/Smad2 signaling pathway [38]. Notably, the cardiac tissues of STZ‐treated rats in this study exhibited a marked increase in lipid peroxides, NF‐*κ*B activity, and inflammatory cytokines, alongside a significant decrease in SOD activity, GSH, and Nrf2 levels. Chronic low‐grade inflammation is now recognized as a key driver of DCM progression, with persistent hyperglycemia triggering excessive immune cell recruitment and activation. These alterations may result in a hostile myocardial microenvironment that promotes cardiac damage [39]. The inflammatory response in DCM is characterized by a feedback loop between regulated cell death and inflammatory signaling, which amplifies myocardial injury and accelerates disease progression [40]. Proinflammatory cytokines such as IL‐1*β*, TNF‐*α*, and IL‐6 activate downstream pathways including NF‐*κ*B and the NLRP3 inflammasome, contributing to fibrosis and heart failure [41]. Additionally, present data regarding the upregulation of Bax and elevated activity of CASP‐3, coupled with the decreased protein levels of Bcl‐2, further substantiates the involvement of intrinsic cell death in mediating STZ‐induced cardiac injury in diabetic rats.

Notably, the pathogenesis of DCM involves complex interactions between multiple cardiac cell types, not just cardiomyocytes. Cardiac nonmyocytes (e.g., fibroblasts, vascular smooth muscle cells, endothelial cells, and immune cells) play critical roles in disease development and progression [42]. Fibroblasts contribute to myocardial fibrosis through excessive extracellular matrix deposition, whereas endothelial cell dysfunction promotes microvascular rarefaction and impaired oxygen delivery [43]. Immune cells, particularly macrophages, orchestrate the inflammatory response through cytokine secretion and intercellular signaling. This cellular heterogeneity underscores the importance of multitargeted therapeutic approaches for DCM. SIRT1 is expressed in multiple cardiac cell types, and its activation by NAR likely occurs across several cellular compartments. In cardiomyocytes, SIRT1 activation protects against hyperglycemia‐induced mitochondrial dysfunction, apoptosis, and oxidative stress through the SIRT1/AMPK/PGC1*α* axis. In cardiac fibroblasts, SIRT1 upregulation inhibits the phenotypic transformation of fibroblasts into myofibroblasts via the Akt/*α*‐SMA pathway, thereby attenuating cardiac fibrosis [44]. In immune cells, particularly macrophages, SIRT1 regulates the M1/M2 polarization balance. SIRT1 activation promotes M2 polarization and reduces inflammatory responses, whereas SIRT1 downregulation is associated with M1 macrophage polarization and enhanced inflammation [45].

Previous studies have demonstrated that flavonoids, including NAR, can attenuate STZ‐induced cardiac oxidative stress and inflammation in murine models [8, 9, 11]. Oral administration of naringin leads to the detection of NAR metabolites in the heart tissue of rats, confirming its bioavailability in the myocardium [46]. SIRT1 mediates these effects by promoting the synthesis of GSH and Phase II antioxidant enzymes (SOD and catalase) through deacetylation and activation of Nrf2 and FOXO1 [47, 48]. SIRT1 also directly inhibits inflammatory cytokine production and decreases ROS generation by deacetylating NF‐*κ*B [49, 50], suppresses intrinsic cell death by downregulating Bax through p53 deacetylation [51], and enhances cardiac contractility by regulating intracellular calcium levels [52]. Consistent with prior studies [53–55], STZ significantly diminished SIRT1 levels in the present study, accompanied by increased Nrf2, NF‐*κ*B, Smad2, and FOXO1. This disruption in SIRT1 signaling likely contributes to cardiac oxidative damage, as it suppresses antioxidant mechanisms while activating inflammatory, fibrotic, and apoptotic pathways. Cardiac SIRT1 depletion alone is sufficient to elicit a DCM‐like phenotype with cardiomyocyte loss, oxidative stress, inflammation, hypertrophy, and fibrosis [56]. NAR treatment restored SIRT1 levels and decreased these transcription factors, effects that were negated by EX‐527. Other flavonoids (e.g., ellagic acid and quercetin) have similarly been shown to activate SIRT1 in various tissues [57, 58].

Part of NAR′s cardioprotective actions may also stem from its hypoglycemic and insulin‐releasing properties. Hyperglycemia plays a crucial role in mediating oxidative stress, inflammation, fibrosis, and apoptosis in diabetic hearts. NAR lowers fasting blood glucose in both basal and diabetic conditions and stimulates insulin release exclusively in diabetic rats. This is mediated through several complementary mechanisms. First, AMPK‐dependent GLUT4 translocation to the plasma membrane increases skeletal muscle glucose uptake [59]. Second, NAR reduces pyruvate transport into mitochondria and inhibits hepatic gluconeogenesis, similar to metformin [60, 61]. Third, NAR protects pancreatic *β* cells from STZ‐induced apoptosis and enhances glucose‐stimulated insulin secretion via estrogen receptor *β* activation [62]. Fourth, it inhibits intestinal glucose absorption [63]. SIRT1 contributes to these effects by enhancing GLUT expression, boosting insulin signaling, and stimulating AMPK [63]. The hypoglycemic and insulin‐releasing effects of NAR were inhibited by EX‐527, confirming SIRT1 dependence; however, the tissue site of this activation (liver, skeletal muscle, or *β* cells) remains unclear and requires further investigation. Flavonoids have been shown to elevate SIRT1 activity in hearts and other tissues [64–66].

This study has several novel contributions. First, it demonstrates that NAR′s cardioprotective effects are mediated through SIRT1 activation, confirmed by EX‐527. Second, unlike previous studies focusing on single pathways, SIRT1 activation by NAR orchestrates a coordinated multitargeted response. Translational application faces challenges including cardiac‐specific targeting, NAD availability optimization, and limited clinical studies [67]. Nonetheless, these findings support NAR′s potential as a nutraceutical SIRT1 activator for diabetic cardiac complications. Although present preclinical findings demonstrate the therapeutic potential of NAR as a SIRT1 activator in diabetic cardiac injury, translation to clinical practice faces several challenges. SIRT1 expression is significantly reduced in the cardiomyocytes of diabetic patients and is associated with multiple signaling pathways (e.g., AMPK/SIRT1 and FoxO3a/SIRT1). Also, SIRT1 serves as a prognostic biomarker in heart failure and diabetic patients [67]. However, achieving cardiac‐specific targeting, optimizing NAD availability, and translating preclinical findings into clinical practice remain significant hurdles [67]. Clinical studies on SIRT1 activators remain limited in number and sample size. Nevertheless, present findings contribute to this translational effort by providing mechanistic evidence for NAR as a SIRT1 activator with multitargeted cardioprotective effects, supporting its potential as a nutraceutical approach for diabetic cardiac complications.

Several limitations of this study should be acknowledged. The STZ‐induced diabetic rat model, while widely used, does not fully replicate the clinical definition of DCM due to the presence of hypertension. The absence of echocardiography precluded direct assessment of cardiac function. The exclusive use of male rats limits generalizability to females, and the STZ model primarily represents T1DM, with applicability to T2DM remaining unclear. Additionally, cardiac hypertrophy was assessed using H/B ratio rather than heart‐to‐tibia ratio, and histopathological evaluation was qualitative rather than quantitative. The 8‐week treatment duration may not capture the full chronic disease spectrum. Despite these limitations, our findings provide robust mechanistic evidence for SIRT1‐mediated cardioprotection by NAR in diabetic cardiac injury.

## 5. Conclusion

In conclusion, NAR appears to confer cardioprotection in STZ‐induced DCM, likely through activation of myocardial SIRT1, which orchestrates a multitargeted response including ameliorating hyperglycemia and dyslipidemia, suppressing oxidative stress via the Nrf2/GSH/SOD axis, inhibiting inflammation through NF‐*κ*B/TNF‐*α*/IL‐6 downregulation, attenuating fibrosis by TGF‐*β*/Smad2 modulation, and reducing apoptosis via Bax/Bcl‐2/CASP‐3 pathway normalization. The complete abolition of benefits by EX‐527 confirms SIRT1 as the nodal regulator. These findings suggest that NAR may represent a promising therapeutic candidate, warranting clinical evaluation for diabetic cardiovascular complications.

## Author Contributions

All authors made a significant contribution to the work reported, whether that is in the conception, study design, execution, acquisition of data, analysis and interpretation, or in all these areas, and took part in drafting, revising, or critically reviewing the article.

## Funding

This study was funded by the Science and Technology Development Program of Jinan Health Commission (2025301011).

## Disclosure

The authors gave final approval of the version to be published, have agreed on the journal to which the article has been submitted, and agree to be accountable for all aspects of the work.

## Ethics Statement

The experimental design and procedures were performed based on the international guidelines related to animal care and the National Institutes of Health (NIH). The handling, treatment, and sacrifice of animals and all procedures related to this investigation were performed according to the ARRIVE guidelines. Approval was granted by the Ethical Committee of the Fourth People′s Hospital of Jinan (No.: LL20250027).

## Consent

The authors have nothing to report.

## Conflicts of Interest

The authors declare no conflicts of interest.

## Supporting information


**Supporting Information** Additional supporting information can be found online in the Supporting Information section. The following supporting information can be downloaded at the journal′s website. Table S1: Primer sequences used for quantitative real‐time PCR (qRT‐PCR) analysis of target genes in cardiac tissue. This table details the forward and reverse primer sequences for *β*‐actin (endogenous control), Bax, CASP‐3, Bcl‐2, NF‐*κ*B, SIRT1, FOXO1, and Nrf2, as referenced in the Gene Expression Analysis subsection of the Materials and Methods.

## Data Availability

The data that support the findings of this study are available from the corresponding authors upon reasonable request.
