# Supplementary material for: Naringenin Attenuates Diabetic Cardiac Injury via SIRT1 Activation in Streptozotocin‐Induced Rats
Source: Cardiovasc Ther. 2026 Oct 9;2026:9581618. doi: 10.1155/cdr/9581618 (PMC13651716; doi:10.1155/cdr/9581618)
Supplement: Supplementary file 1 [file CDR-2026-9581618-s001.docx]

**Supplementary Table 1. Primer sequences.**

| Primer | Sequence |
| --- | --- |
| β-actin forward | 5′-TGTCACCAACTGGGACGATA-3′ |
| β-actin reverse | 5′-GGGGTGTTGAAGGTCTCAAA-3′ |
| BAX forward | 5′- ATCCAAGACCAGGGTGGCTG-3′ |
| BAX reverse | 5′-CACAGTCCAAGGCAGTGGGA-3′ |
| CASP3 forward | 5′-GAGCTTGGAACGCGAAGAAA-3′ |
| CASP3 reverse | 5′-GTGGAACTGAGACACGTAGC-3′ |
| BCL-2 forward | 5′-TATATGGCCCCAGCATGCGA-3′ |
| BCL-2 reverse | 5′-GGGCAGGTTTGTCGACCTCA-3′ |
| NF-κB forward | 5′-CACGCTTCTGGGCCTGTT-3′ |
| NF-κB reverse | 5′-TGAGACAGCACGTGGATGCT-3′ |
| SIRT1 forward | 5′-GAGTGTGCTGGAGGATCTG-3′ |
| SIRT1 reverse | 5′-TGCTCTGATTTGTCTGGTGT-3′ |
| FOXO1 forward | 5′-CGAGTGGATGGTGAAGAGTG-3′ |
| FOXO1 reverse | 5′-CGAATAAACTTGCTGTGTAGGG-3′ |
| Nrf2 forward | 5′-CGAACAGCACCTCTTGGTCTG-3′ |
| Nrf2 reverse | 5′-CACAGACGCAGAGGATTACTGCGA-3′ |
